# Introduction of laparoscopic nephrectomy for autosomal dominant polycystic kidney disease as the standard procedure

**DOI:** 10.1007/s00423-022-02737-9

**Published:** 2023-01-05

**Authors:** M. N. Thomas, R. R. Datta, R. Wahba, D. Buchner, C. Chiapponi, C. Kurschat, F. Grundmann, A. Urbanski, S. Tolksdorf, R. Müller, J. Henze, V.-M. Petrescu-Jipa, F. Meyer, C. J. Bruns, D. L. Stippel

**Affiliations:** 1 Department of General-, Visceral-, Tumor- and Transplantation Surgery, University of Cologne, Faculty of Medicine and University Hospital of Cologne, University Hospital of Cologne, Cologne, Germany; 2 Department II of Internal Medicine and Center for Molecular Medicine Cologne, University of Cologne, Faculty of Medicine and University Hospital of Cologne, Cologne, Germany; 3 Department of Radiology, University of Cologne, Faculty of Medicine and University Hospital of Cologne, University Hospital of Cologne, Cologne, Germany; 4 Department of Transfusionsmedizin, University of Cologne, Cologne, Germany

**Keywords:** Laparoscopic nephrectomy, ADPKD, Kidney transplantation

## Abstract

**Purpose:**

Autosomal dominant polycystic kidney disease (ADPKD) is a common hereditary disorder and accounts for 5–10% of all cases of kidney failure. 50% of ADPKD patients reach kidney failure by the age of 58 years requiring dialysis or transplantation. Nephrectomy is performed in up to 20% of patients due to compressive symptoms, renal-related complications or in preparation for kidney transplantation. However, due to the large kidney size in ADPKD, nephrectomy can come with a considerable burden. Here we evaluate our institution’s experience of laparoscopic nephrectomy (LN) as an alternative to open nephrectomy (ON) for ADPKD patients.

**Materials and methods:**

We report the results of the first 12 consecutive LN for ADPKD from August 2020 to August 2021 in our institution. These results were compared with the 12 most recent performed ON for ADPKD at the same institution (09/2017 to 07/2020). Intra- and postoperative parameters were collected and analyzed. Health related quality of life (HRQoL) was assessed using the SF36 questionnaire.

**Results:**

Age, sex, and median preoperative kidney volumes were not significantly different between the two analyzed groups. Intraoperative estimated blood loss was significantly less in the laparoscopic group (33 ml (0–200 ml)) in comparison to the open group (186 ml (0–800 ml)) and postoperative need for blood transfusion was significantly reduced in the laparoscopic group (*p* = 0.0462). Operative time was significantly longer if LN was performed (158 min (85–227 min)) compared to the open procedure (107 min (56–174 min)) (*p* = 0.0079). In both groups one postoperative complication Clavien Dindo ≥ 3 occurred with the need of revision surgery. SF36 HRQol questionnaire revealed excellent postoperative quality of life after LN.

**Conclusion:**

LN in ADPKD patients is a safe and effective operative procedure independent of kidney size with excellent postoperative outcomes and benefits of minimally invasive surgery. Compared with the open procedure patients profit from significantly less need for transfusion with comparable postoperative complication rates. However significant longer operation times need to be taken in account.

## Introduction

ADPKD is a progressive disorder, which is characterized by multiple bilateral cysts of the kidney parenchyma leading to loss of kidney function and eventually to the requirement of kidney replacement therapy (KRT) in the majority of patients [1–3]. ADPKD is mostly associated with an asymptomatic course, with first symptoms appearing in the third decade of life. It is primarily caused by a mutation in one of two genes, PDK1 and PDK2 leading to abnormal cilia-mediated signaling pathways and the formation and expansion of cysts [4]. ADPKD accounts for approximately 5–10% of all causes of kidney failure requiring KRT.

Patients with ADPKD frequently show symptoms, partly due to the large size of the polycystic kidneys. Compressive symptoms include abdominal pain and distension, nausea, back pain and early satiety. Furthermore, patients can get symptomatic exhibiting gross hematuria, pain exacerbation related to acute hemorrhagic cysts, recurrent urinary tract infections due to infected cysts, nephrolithiasis, and therapy-refractory hypertension.

Approximately 20% of patients with ADPKD will be treated with nephrectomy because of the prementioned complications. Another frequent indication for unilateral nephrectomy consists in creating sufficient space while performing or in preparation of a planned kidney transplantation.

In most European centers, cystnephrectomy is performed by open laparotomy over a large midline incision. These procedures are often associated with a significant complication rate and associated morbidity (38%) and mortality (3%) for the patients [5, 6].

Since its first description in 1996, LN for ADPKD has been reported in many centers, demonstrating its feasibility and underlining the known benefits of this minimal approach as shorter hospital stay, decreased morbidity and quicker recovery from the procedure [5, 7–10]. However, in most European countries, LN does not represent the standard of care for this group of patients. Due to the sheer size of the kidneys laparoscopic preparation can be challenging and specimen extraction can jeopardize the benefits of the laparoscopic approach.

In our institution, cystnephrectomies were conducted in the past by open laparotomy with a large subcostal incision and retroperitoneal preparation. Since August 2020, we introduced LN at our institution and since established this technique as the new standard of care for surgical treatment of ADPKD-patients.

Here, we describe our technique and surgical outcomes of the first 12 consecutively performed LN for ADPKD and compare these results to the last 12 consecutively performed open cystnephrectomies at our institution.

## Material and methods

All consecutive laparoscopic cystnephrectomies were performed at the surgical department of the Cologne University Hospital by two experienced surgeons between August 2020 and August 2021.

Informed consent for LN was obtained from each patient. The perioperative and postoperative parameters were compared to the last 12 consecutively ON for ADPKD at our institution performed between September 2017 and March 2020.

**Fig. 1:**
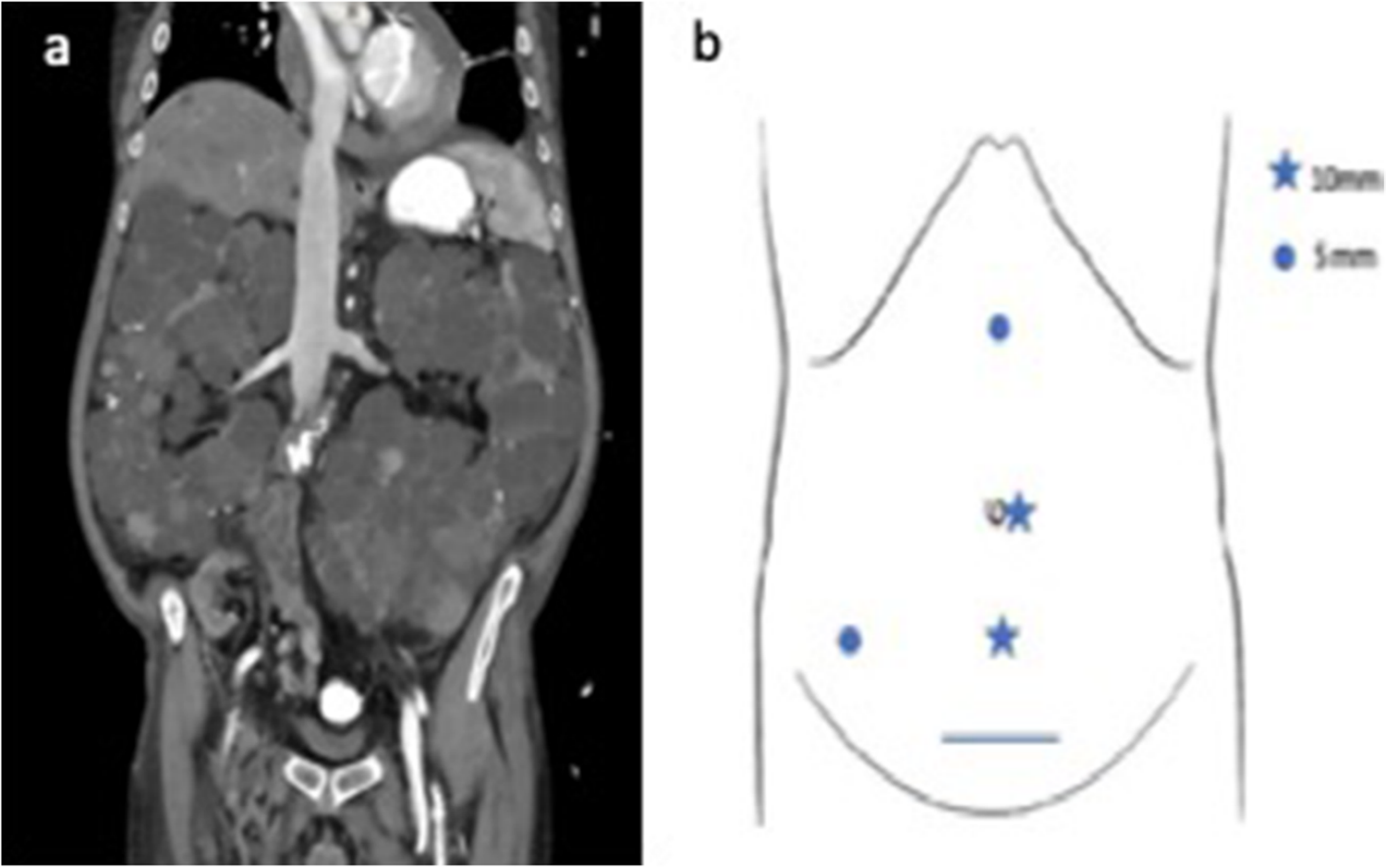
**a** Preoperative CT scan after kidney transplantation and before staged bilateral cystnephrectomy. **b** Port placement for right sided LN

All patients underwent abdominal CT or MRI scan during preoperative evaluation (Fig. 1a) and received cefuroxim as a perioperative antibiotic treatment.

For unilateral LN patients were placed in a lateral decubitus position with the ipsilateral side up and a 30° backward inclination. The umbilical port was used as camera port and 3 further trocars were placed circumferentially (Fig. 1b).

**Fig. 2:**
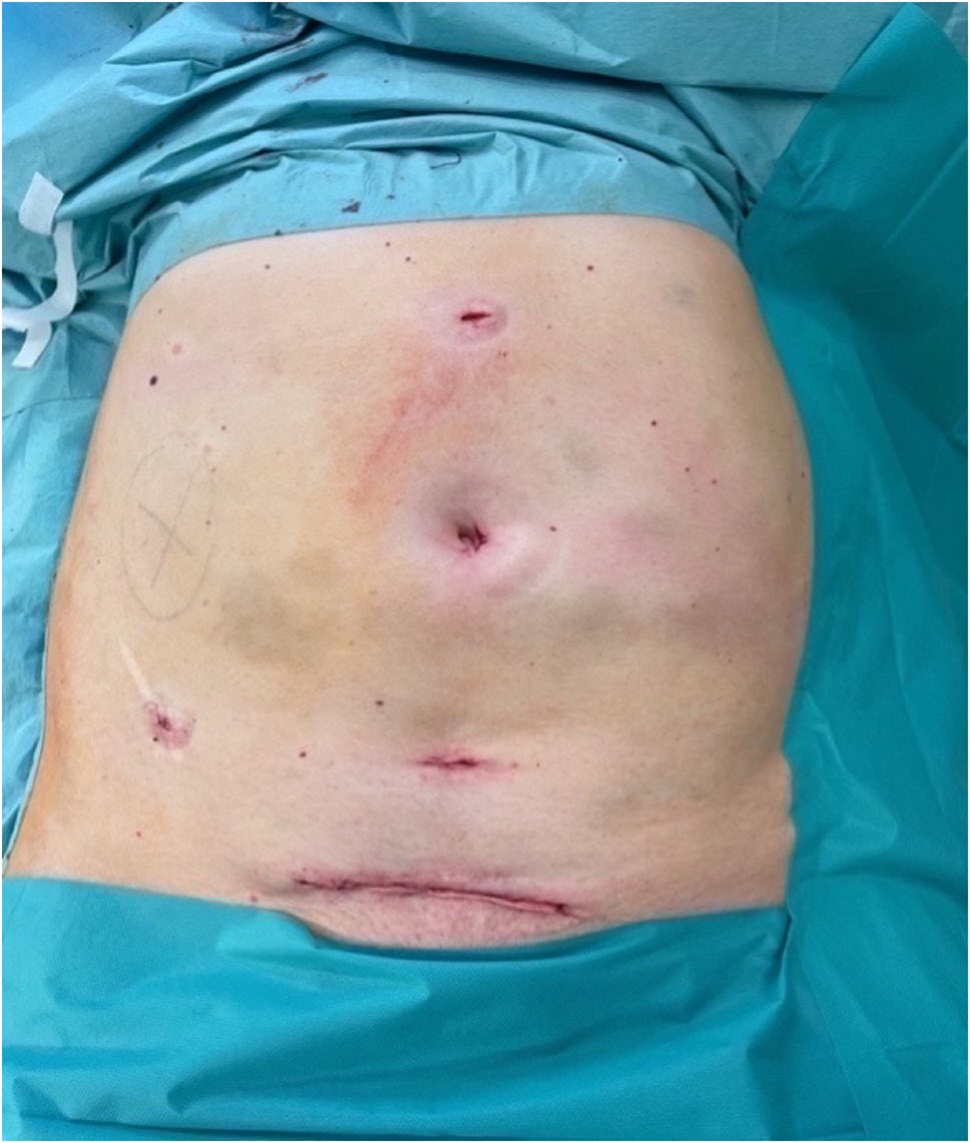
Postoperative situs after laparoscopic right sided nephrectomy

After installation of the capnoperitoneum the colon was mobilized and the ureter was localized and clipped. Hereafter, the largest cysts were disrupted and the cyst fluid was aspirated in order to minimize kidney volume followed by the localization of the vascular pedicle which was subsequently dissected with a vascular stapler (Echelon Flex, Ethicon/Johnson & Johnson). After devascularization, the upper pole and the lateral retroperitoneal fixation of the kidney was mobilized and a large recovery bag (volume 2,5 l) was placed in the abdominal cavity in order to extract the kidney in piecemeal technique through an additional supra-pubic incision (Fig. 2). In cases where unilateral cystnephrectomy was performed before kidney transplantation, the paramedian incision used for subsequent kidney transplantation was used to extract the specimen.

In order to evaluate postoperative health-related quality of life (HRQoL) the internationally validated SF-36 questionnaire was mailed to all patients 3 months after surgery. This questionnaire consists of 36 items combined into 8 scales: physical function, role function limited by physical problems, bodily pain, general health, vitality, social functioning, role function limited by emotional problems, and mental health.

Subscale scores are further transformed to a 0–100 scale, with 0 representing the worst well-being and 100 representing the greatest well-being. The SF-36 is well established and validated to measure postoperative recovery after various surgical procedures [11, 12].

Electronic and paper data of the University Hospital of Cologne were retrospectively collected and analyzed. Written informed consent was obtained from every patient before performing the laparoscopic procedure.

Statistical analysis were performed using SPSS 28.0.1 for Windows (IBM, Armonk, NY, USA) and GraphPad Prism 4 (GraphPad Software, Inc., La Jolla, CA, USA). Besides descriptive statistics means were compared with student t-test. Categorial variables were compared using the Fisher’s exact test. A p-value of < 0.05 was considered to indicate statistical significance.

The manuscript was submitted to the local ethics committee which stated that this study is exempt from applying for ethical approval, as under German law no separate ethics application and statement of ethical approval by the local ethics committee are required for performing purely retrospective clinical studies.

## Results

Twelve consecutive LN for ADPKD were performed from August 2020 to August 2021 at our department by two experienced transplant surgeons. These operations were performed in nine different patients. In one patient, simultaneous bilateral nephrectomy was performed and in two patients staged bilateral nephrectomy was performed. Six patients received unilateral LN. All analyzed patients in the ON group received unilateral cystnephrectomy. All patients had either received a kidney transplant or were on dialysis.

In the laparoscopic group, eight nephrectomies were performed due to pain and abdominal discomfort. Two patients suffered from recurrent hematuria with the need of repeated blood transfusion. One nephrectomy was performed during living donation kidney transplantation and one in preparation for future renal transplantation. In the ON group, four nephrectomies were performed during simultaneous kidney transplantation, four due to recurrent infections and three due to symptomatic cysts. In one patient, unilateral nephrectomy was performed in preparation for future kidney transplantation (Table 1).

**Table 1:** Patients characteristics

	Laparoscopic	Open	*P* value
No. of patients/procedures (*n*)	9/12	12/12	n.a
Age, mean (years)	60 (39–68)	55 (34–68)	n.s
Male gender, *n* (%)	6 (67%)	9 (75%)	n.s
BMI, mean	25 (19–33)	27 (21.5–32.4)	n.s
Kidney volume (ml)	3795 (1255–8253)	2641 (625–5695)	n.s
Indication for surgery (*n*)			
Pain	8	3	n.a
Macrohaematuria	2	0	n.a
Infection	0	4	n.a
Pretransplant	1	1	n.a
During kidney TX	1	4	n.a
Dialysis at time of nephrectomy (*n*)	8	3	n.s

*na* not applicable, *ns* not significant, *BMI* body mass index, *ml* milliliters, *n* number, *()* numbers in parentheses are the range

At the time of nephrectomy, eight patients in the laparoscopic group were on dialysis whereas only three in the open procedure group (Table 1).

**Fig. 3:**
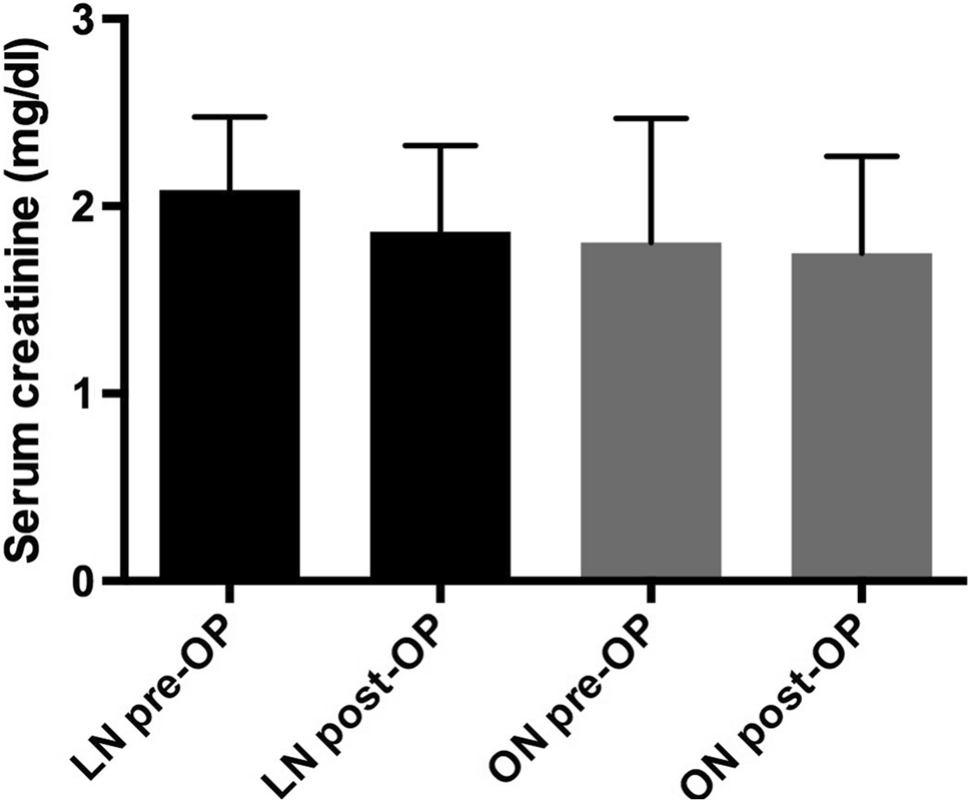
Serum creatinine values pre- and post-cystnephrectomy

Postoperative creatinine levels measured on postoperative day 7 showed stable values in both groups, excluding patients where simultaneous kidney transplantation was performed directly after LN and those on preoperative dialysis (Fig. 3). Five patients in the open procedure group underwent prior kidney transplantation and four patients in the laparoscopic group.

Cystnephrectomies were often combined with other procedures. In the laparoscopic group one hernia repair after kidney transplantation was performed, one fenestration of a clinically relevant lymphocele was conducted and one simultaneous kidney transplantation (living-donation) was performed. In the ON group, 4 simultaneous kidney transplantations were performed.

The mean age of patients was 60 years (range 39–68 years) in the laparoscopic group respectively 55 years (range 34–68 years) in the ON group. Mean BMI was 25 (range 19–33) in the laparoscopic group compared to 27 (21.5–32.4) in the open group. Sixty-seven percent of the patients were male in the laparoscopic group and 75% in the ON group (Table 1).

Mean preoperative volumetric analyses using abdominal CT-scans of the resected kidneys was 3795 ml (range 1255–8253 ml) in the laparoscopic group and 2641 ml (range 625–5695 ml) in the open procedure group. Mean operative time was with 158 min (range 85–227 min) in the laparoscopic group significantly longer (*p* = 0.0079) if compared to the mean of 107 min (range 56–174 min) in the open group. The mean weight of the resected kidney was 1589 g (832–2670) in the laparoscopic group compared to 1917 g (range 420–2775 g) in the open cystnephrectomy group (Table 2).

**Table 2:** Perioperative surgical data

	Laparoscopic	Open	*P* value
Mean operative time (min)	158 (85–227)	107 (56–174)	*p* = 0.0079
Kidney weight (g)	1589 (832–2670)	1917 (420–2775)	n.s
Blood loss (ml)	33 (0–200)	186 (0–800)	*p* = 0.0330
Blood transfusion (rcc)	0.4 (0–2)	2.2 (0–10)	*p* = 0.0462
Mean length of hospital stay (days)	10 (6–35)	16 (4–56)	n.s
Side			
Right	5	6	n.a
Left	1	6	n.a
Simultaneous bilateral	1	0	n.a
Staged bilateral	2	0	n.a
Complications (Clavien-Dindo ≥ 3), *n* (%)	1/12 (8,3%)	1/12 (8,3%)	n.s
Conversion to open surgery, *n* (%)	0/12 (0%)	–	n.a

*ns* not significant, *min* minutes, *g* grams, *ml* milliliters, *rcc* red cell concentrates, *n* number, *()* numbers in parentheses are the range

**Fig. 4:**
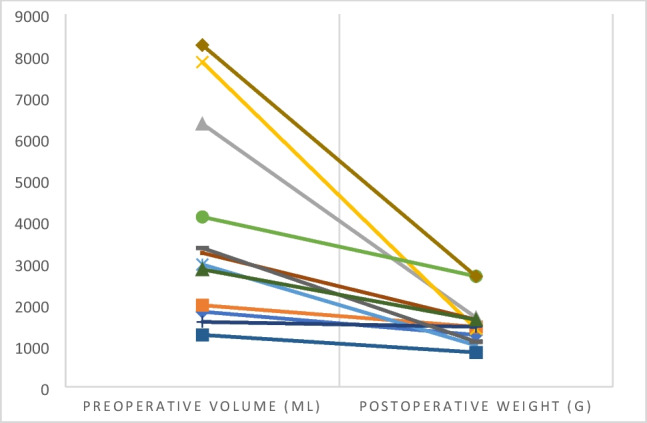
Intraoperative specimen volume reduction of laparoscopic performed cystnephrectomies

Mean volume reduction through intraoperative fluid aspiration of the largest cysts in order to facilitate the operative procedure in the laparoscopic group was 49% (range 8–82%) (Fig. 4).

**Fig. 5:**
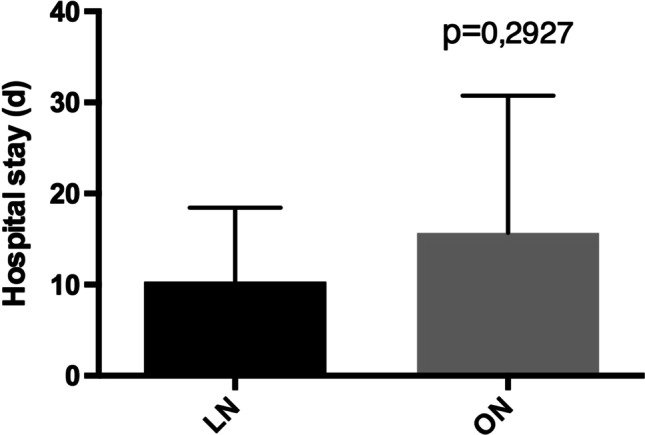
Postoperative hospital stay

The mean postoperative hospital stay was 10 days (range 6–35 days) in the laparoscopic group compared to 16 days (4–56) days in the open procedure group (Table 2) without showing statistical significance (*p* = 0.2927)(Fig. 5).

Neither in the LN nor in the ON group intraoperative complications occurred. Especially in the laparoscopic group no conversion to open surgery needed to be performed.

The only postoperative complication Clavien Dindo ≥ 3 in the LN group occurred in one patient where bilateral nephrectomy was performed. On postoperative day 1, occlusion of the hemodialysis shunt occurred and needed vascular intervention. In the ON group, one postoperative abdominal wound dehiscence after laparotomy occurred and needed repeat surgery.

All pathological specimens showed benign findings of cystic kidneys.

**Fig. 6:**
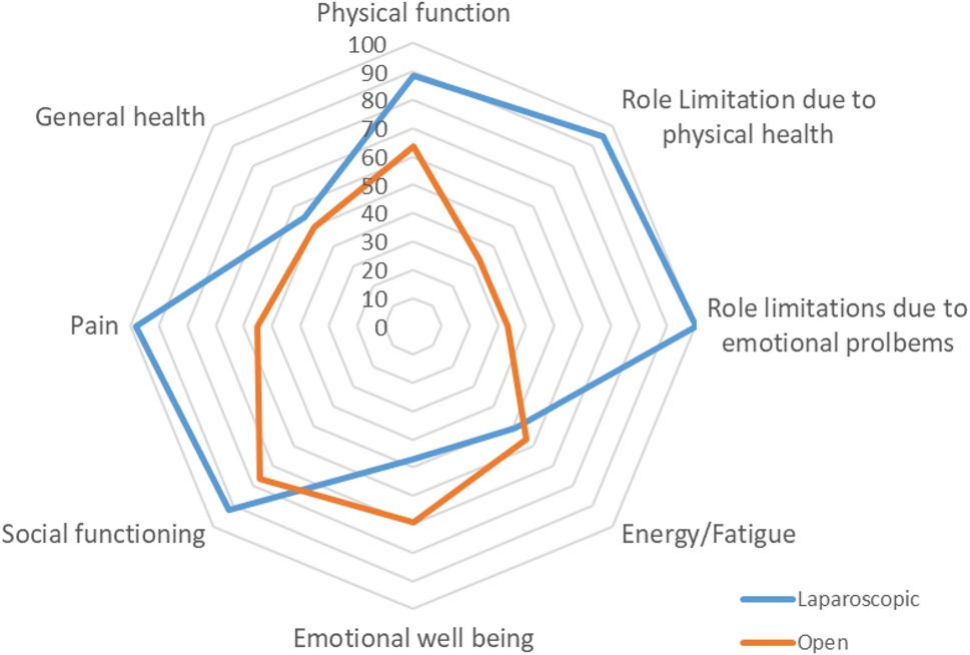
Postoperative HrQoL according to SF-36 questionnaire

SF36 health survey questionnaire revealed excellent postoperative quality of life (Fig. 6) for laparoscopically operated patients with markedly postoperative limitations in the conventionally open operated group especially regarding pain, social function, and role limitations due to physical health or emotional problems.

Since the evaluation of these first LN for ADPKD all following cystnephrectomies until September 2022 were done in the same laparoscopic fashion without a single conversion to open surgery or Clavien Dindo ≥ 3 morbidity.

## Discussion

Approximately 20% of ADPKD patients will require nephrectomy due to compressive syndromes, renal-related complications or in order to generate space during or before kidney transplantation [13]. In the past, ON was the standard procedure for unilateral or bilateral nephrectomy in ADPKD patients. The operation was performed over a large midline incision. This procedure however is associated with high morbidity and mortality rates [5, 13].

Laparoscopic nephrectomy was first described in 1990 by Claymann et. al. [14] and since then has become a widely accepted procedure. As a consequence, larger kidneys have been approached and in 1996 Clayman et al. [8] was again the first to describe the first successful laparoscopic cystnephrectomy. From here, laparoscopic cystnephrectomy has been introduced in many centers demonstrating its feasibility [5, 9, 10]. However, in most European centers, ON for ADPKD patients still remains the standard of care because of technical challenges and space constrains in the abdominal cavity due to large kidney size as well as occasional intense perinephric inflammatory reaction as a consequence of earlier cyst infections or hemorrhage [15].

LN for ADPKD is accompanied by typical advantages of the minimal invasive approach. Guo et al. [5] could demonstrate in his meta-analysis of open vs. laparoscopic nephrectomy for patients with ADPKD including 7 studies with a total of 195 patients (118 laparoscopic nephrectomy/77 ON) that the laparoscopic group benefits from a shorter hospital stay, less estimated blood loss, and a lower need of transfusion. In addition, the laparoscopic group had significantly less overall complication rate. However, laparoscopic nephrectomy was associated with a significantly longer operation time compared to the open procedure.

In our study we could demonstrate comparable results regarding the need for transfusion and estimated intraoperative blood loss. In addition, we could observe a significant longer operative time in the laparoscopic group while showing a trend towards shorter hospital stay without reaching statistical significance probably due to the small group size and the group inhomogeneity. In our study, however the open and laparoscopic group showed comparable results regarding major complication rates.

The demonstrated benefits of the minimal invasive approach can be at risk, if intact specimen removal is performed over a large incision conditional to the sheer size of the polycystic kidneys [7]. Therefore, we performed intraoperative disruption of the largest cysts and aspiration of the cyst-fluid as well as morcellation of the specimen in a large recovery bag. As visualized in Fig. 3, especially in giant polycystic kidneys (volume > 5000 ml), intraoperative weight reduction was more pronounced if compared to smaller kidneys. We think that adequate volume reduction through aspiration of the largest cysts is one of the key steps in LN for ADPKD in order to facilitate further preparation. In addition, with this technique the suprapubic incision can be kept minimal (Fig. 2), hereby reducing the risk of postoperative wound related complications risking the demonstrated advantages of the minimal invasive approach.

The conversion rate to open surgery varies in the literature between 0 and 22% [9, 10, 16, 17]. Because of the large size of the kidneys and the hereby limited available space during the laparoscopic procedure, incidental injury of adjacent organs or technical irresectability can occur during the procedure [16] leading to conversion to open surgery. Ivey et al. [18] could demonstrate in a series of 399 laparoscopic renal surgeries that large specimens defined as > 1500 g had a significantly higher incidence of conversion to open surgery (42% vs. 8%, *p* < 0.001). In addition, nephrectomy in ADPKD is generally associated with a high learning curve.

In our institution, living kidney donation is performed in a hand-assisted retroperitoneoscopic (HARP) procedure. So far, over 400 HARP-procedures could be safely performed without the need of conversion to open surgery. This emphasizes the broad experience of LN in our institution and explains the low complication and conversion rate experienced in our series of LN for ADPKD if compared to the above-mentioned literature. With the acquired laparoscopic experience from the HARP-procedure, implementation of laparoscopic cystnephrectomy could be established in our institution without the previously described high learning curve [16].

HRQoL is an important outcome measure for minimal invasive techniques and has been evaluated in many surgical procedures showing increased QoL for laparoscopic procedures especially in the early postoperative phase if compared to open procedures [12, 19–21].

In our study, postoperative HRQoL showed better results for the laparoscopic group especially regarding pain, social function, and role limitations due to emotional problems and physical health. These results however need to be interpreted with restraint due to the inhomogeneity of the analyzed groups.

This study has some limitations. Its retrospective nature and the relatively small and inhomogeneous group sizes.

## Conclusion

LN for ADPKD can safely be introduced as the standard procedure in a center with experience in LN. This approach allows a significant reduced need of postoperative transfusions with excellent postoperative quality of life for ADPKD patients while experiencing comparable postoperative complication rates.
